# Endoscopy-assisted vitrectomy vs. vitrectomy alone: comparative study in complex retinal detachment with proliferative vitreoretinopathy

**DOI:** 10.1186/s40942-020-00238-9

**Published:** 2020-07-29

**Authors:** Flavio A. Rezende, Natalia Vila, Emmanouil Rampakakis

**Affiliations:** 1 Department of Ophthalmology (Centre Universitaire d’Ophtalmologie), Maisonneuve Rosemont Hospital, CIUSS de l’est d’Ile de Montréal, Montreal, QC Canada; 2 St Paul’s Eye Unit, Royal Liverpool University Hospital NHS Trust, Prescot St, Liverpool, L7 8XP UK; 3 Medical Affairs, JSS Medical Research, Montreal, Canada

**Keywords:** Endoscopy-assisted vitrectomy, Endoscopy-guided vitrectomy, Endoscopic vitrectomy, Proliferative vitreoretinopathy, Retinal detachment

## Abstract

**Background:**

Recurrent retinal detachment (RD) is still a widespread event despite the therapeutic options available. Proliferative vitreoretinopoathy (PVR) is one of the main causes of redetachment. Little is known about the use of endoscopy-assisted vitrectomy (E-PPV) in complex recurrent RD with PVR. The purpose of this study was to identify the potential advantages of E-PPV in complex RD with PVR compared with pars plana vitrectomy (PPV) alone.

**Methods:**

Single-center, retrospective, observational, descriptive study. The medical records of 293 patients were reviewed. Patients who underwent PPV for complex rhegmatogenous RD and associated PVR between 2009 and 2017 were included. Patients with diabetic tractional RD, trauma, uveitis or detachment postendophthalmitis were excluded. After 2013, an endoscopic visualization system was used in a nonrandomized fashion at the surgeon’s discretion. Outcome measures (reattachment rate, number of surgeries, lens status, PVR stage, intraocular pressure, phthisis rate) were compared between the E-PPV and PPV-only groups with independent samples t-tests (continuous variables) and Fisher’s exact test (categorical variables), as well as time-adjusted analyses. Postoperative time to retinal redetachment was assessed with Kaplan–Meier survival analysis.

**Results:**

One hundred one eyes from 100 patients met the inclusion criteria. The mean participant age was 63.3 years old (95% CI 60.4–66.1 years), without a significant difference between groups. E-PPV was performed in 36.6% (n = 37) of eyes, and 63.4% (n = 64) underwent PPV only. The mean follow-up was significantly longer in the PPV-only group (31.9 vs. 21.1 months; p = 0.021). Upon adjustment for follow-up duration, the mean number of surgeries was significantly lower in the PPV-only group (2.6 vs. 4.3 number of surgeries; p < 0.001) than in the E-PPV group. A significantly higher risk for redetachment was observed in the PPV-only group (HR [95% CI] 4.1 [1.4–11.8]) than in the E-PPV group (p = 0.037). The evolution to phthisis was 7% (n = 4) in the PPV-only group and 2.7% (n = 1) in the E-PPV group (p > 0.05).

**Conclusions:**

Compared to PPV alone, endoscopy-assisted vitrectomy seems to be advantageous in achieving better reattachment rates in complex RD with advanced PVR. Endoscopic visualization allows a thorough examination and extensive anterior PVR and vitreous base dissection.

## Background

Recurrent rhegmatogenous retinal detachment (RRD) after surgical repair remains a relatively widespread event despite the therapeutic options currently available. Proliferative vitreoretinopoathy (PVR) is one of the main causes of redetachment and occurs in 8–10% of patients undergoing primary repair [1–4]. The search for better anatomical outcomes has been investigated by comparing or combining surgical techniques [5, 6]. Little is known in the literature about the addition of endoscopy when performing a vitrectomy in recurrent retinal detachment, especially in complex detachments with PVR [7–12].

Vitreoretinal surgery is undergoing an era of technification. Endoscopic vitrectomy has changed over the past years, and currently, it can be integrated into digitally assisted visualization systems to facilitate learning curves and ease of use [13]. There is still a need to improve surgical outcomes in complex cases, and endoscopy-assisted vitrectomy could facilitate and address anterior pathology differently in these patients.

The aim of the current study was to compare the efficacy of endoscopy-assisted pars plana vitrectomy (E-PPV) vs. pars plana vitrectomy (PPV) alone in complex RRD with advanced PVR.

## Methods

This was a single-center, retrospective, observational, descriptive study conducted in accordance with the tenets of the Declaration of Helsinki. The protocol was approved by the local Institutional Review Board. Informed consent was waived due to the retrospective nature of the study. Medical records of the Department of Ophthalmology at Maisonneuve-Rosemont Hospital (CIUSS de l’est d’Ile de Montréal, University of Montreal, Montreal, Quebec, Canada) were screened for patients who underwent PPV for RRD associated with PVR between July 2nd, 2009 and January 31st, 2017. A total of 293 surgical reports were reviewed, and patients meeting the following criteria were included in the study: 18 years of age or more, RRD associated with advanced PVR and a minimum follow-up of 6 months post-PPV. The classification of PVR used was Machemer’s proposal [14], and we included the following categories: (PVR-B) wrinkling of the inner retina surface, retinal stiffness, and/or retinal breaks with rolled edges; (PVR-C1) starfolds posterior to the vitreous base (VB); (PVR-C2) confluent starfolds posterior to the VB; (PVR-C3) proliferation under the retina, annular strand near the disc, linear strands or moth-eaten-appearing sheets (anterior or posterior); (PVR-C4) retina contraction inwards at the posterior edge of the VB, with central displacement of the retina, peripheral retina stretched or posterior retina in radial folds; (PVR C-5) anterior contraction on the retina at the vitreous base, presence of ciliary body (CB) detachment and epiciliary membrane, or iris retraction. The RRD with PVR included in this study, which we referred to as “advanced PVR”, had PVR greater than PVR-A (PVR-B and/or C). Participants with diabetic tractional detachment, an exudative component, a history of trauma (blunt trauma or open globe injury), uveitis or endophthalmitis were excluded. After 2013, an endoscopic visualization system (E2 MicroProbe™; EndoOptiks, Little Silver, USA) was used in a nonrandomized fashion at the surgeon’s discretion. Eligible patients were divided into two groups: (1) the endoscopy-assisted PPV (E-PPV) group, who underwent surgery that combined endoscopic visualization with wide-field visualization, and (2) the PPV-alone group, for whom endoscopy was never used during any of the surgical interventions during follow-up. Patients who underwent more than one surgery and for whom endoscopy was used in at least one surgical procedure during follow-up were included in the E-PPV group.

A minimum of four ocular examinations were performed: prior to surgery, postoperatively (day 1), approximately 1 month after surgery (4 to 6 weeks) and at least 6 months after surgery. All examinations included best corrected visual acuity (BCVA), intraocular pressure (IOP) measurement with applanation tonometry (mmHg), slit lamp biomicroscopy and indirect ophthalmoscopy. The primary outcome was the rate of retinal reattachment, and secondary outcomes were the number of surgeries, lens status, PVR stage, IOP and phthisis rate.

Surgical procedures were performed using 23-gauge or 25-gauge (CONSTELLATION^®^ Vision System) platforms under a microscope with a noncontact wide-field visualization system. In the E-PPV group, a 23-gauge or a 20-gauge probe was used in combination with the standard vitrectomy setup. Endoscopy was performed through a 23-gauge trocar or a 20-gauge sclerotomy. Data extracted from the surgical reports were lens status at the end of the procedure, use of perfluorocarbon liquid, membrane peeling (ERM, ILM, PVR), degrees of retinectomy, laser use, retinectomy extension, anterior retinectomy flap trimming, tamponade and ciliary body (CB) status (when available).

### Statistics

Statistical analysis was performed using SPSS software version 24 (Armonk, NY: IBM Corp.). Summary statistics were produced for all study variables, consisting of the mean and 95% confidence intervals (95% CI) for continuous variables, as well as frequency distributions for categorical variables. Patient characteristics and preoperative and intraoperative ocular characteristics in the E-PPV and PPV-only groups were compared using the independent samples t-test for continuous variables and the *Chi*-squared or Fisher’s exact test, as appropriate, for categorical variables. Comparison of the E-PPV and PPV-only groups in terms of postoperative outcomes was conducted using logistic regression (phthisis), generalized linear models (BCVA, IOP), and Poisson regression (number of surgeries) adjusting for duration of follow-up. Postoperative time to retinal redetachment was assessed with Kaplan–Meier survival analysis, and the Greenwood method was used to calculate the 95% CI of the hazard ratio.

## Results

The study included 101 eyes from 100 patients who underwent PPV for RRD associated with PVR. Endoscopy-assisted PPV was performed in 36.6% (n = 37) of the eyes, and the remaining 63.4% (n = 64) underwent PPV only. The demographic characteristics did not show statistically significant differences between groups (Table 1), although the mean age was numerically lower in the PPV-only group than in the E-PPV group (61.9 vs. 65.6). Preoperative ocular characteristics were similar between the groups (Table 2), with the exception of the underlying diagnosis (RRD and PVR, recurrent RD, recurrent RD under oil, recurrent RD postoil removal), which was significantly different (p = 0.016) between groups.

**Table 1:** Patient characteristics

	PPV-only	E-PPV	*p* value
Number of eyes, n (%)	64 (63.4)	37 (36.6)	N/A
Number of patients, n (%)	64 (64.0)	36 (36.0)	N/A
Gender, n (%)^a^			
Male	41 (64.1)	25 (69.4)	0.663
Female	23 (35.9)	11 (30.6)	
Age at surgery (years)			
Mean (95% CI)	61.9 (57.8–66.1)	65.6 (62.9–68.4)	0.217
Eye, n (%)^b^			
OD	37 (57.8)	23 (62.2)	0.834
OS	27 (42.2)	14 (37.8)	

*N/A* not applicable, *PPV* pars plana vitrectomy, *E*-*PPV* endoscopy-assisted PPV

^a^Proportions based on the total number of patients included

^b^Proportions based on the total number of eyes included

**Table 2:** Preoperative ocular characteristics

	PPV-only	E-PPV	*p* value
Baseline BCVA (LogMAR)			
Mean (95% CI)	1.59 (1.36–1.82)	1.60 (1.30–1.91)	0.944
IOP (mmHg)			
Mean (95% CI)	12.3 (10.5–14.0)	11.3 (9.3–13.3)	0.474
Lens status, n (%)^a,b^			
Phakic	18 (32.7)	2 (5.6)	0.002
Pseudophakic	37 (67.3)	34 (94.4)	
Diagnosis, n (%)^a^			
RD + PVR	31 (48.4)	11 (29.7)	0.016
Recurrent RD	21 (32.8)	8 (21.6)	
Recurrent RD under SO	7 (10.9)	12 (32.4)	
Recurrent RD post-SO removal	5 (7.8)	6 (16.2)	

*PPV* pars plana vitrectomy, *BCVA* best corrected visual acuity, *E-PPV* endoscopy-assisted PPV, *IOP* intraocular pressure, *IOL* intraocular lens, *RD* retinal detachment, *PVR* proliferative vitreoretinopathy, *SO* silicone oil

^a^Proportions based on the total number of eyes included

^b^Lens status was missing for nine eyes in the PPV only group and one eye in the endoscopy-assisted PPV group

Over a mean (95% CI) of 31.9 (25.2–38.6) and 21.1 (16.4–25.8) months of follow-up in the PPV-only and E-PPV groups, respectively, the number of surgeries was significantly higher in the E-PPV group, with 4.1 surgeries compared to 2.7 in the PPV-only group (p < 0.001) (Table 4); adjustment for follow-up duration showed similar statistically significant results. Intraoperatively, in the PPV-only group, the 25-gauge vitrectomy platform was predominantly used (94.3%), while in the E-PPV group, the 25-gauge platform was used in 60% of cases and the 23-gauge platform was used in 40% (p < 0.001) (Table 3). The prevalence of PVR types was similar between the groups, except for PVR-C5, which was higher in E-PPV (9.4% vs. 32.4%; p = 0.006). The same group also presented a higher percentage of perisilicone oil proliferation (3.1% vs. 13.5%; p = 0.048). Overall, large retinectomies were warranted in 44.5% of patients undergoing the last surgical procedure. The proportion of retinectomies larger than 270° was similar in both groups; however, 180° retinectomies were performed in 37.8% of the E-PPV group compared to 17.2% of the PPV-only group, and this difference was statistically significant (p = 0.031) (Table 3). The tamponade used in the last surgical procedure was not significantly different (p = 0.119); eyes with silicone oil used as a tamponade did not show differences between groups (p = 0.315) (Table 4). The distribution of intraocular lenses (IOLs) in the posterior capsular bag, IOLs in the anterior chamber, scleral fixated IOLs or aphakia postsurgery was similar between the two groups (p = 0.276).

**Table 3:** Intraoperative ocular characteristics

	PPV-only	E-PPV	*p* value
PVR			
B, n (%)^a^	26 (40.6)	18 (48.6)	0.533
C, n (%)^a^			
1	19 (29.7)	6 (16.2)	0.156
2	15 (23.4)	7 (18.9)	0.803
3	11 (17.2)	6 (16.2)	> 0.999
3-Anterior	4 (6.3)	3 (8.1)	0.705
3-Posterior	7 (10.9)	3 (8.1)	0.742
4	6 (9.4)	3 (8.1)	> 0.999
5	6 (9.4)	12 (32.4)	0.006
Perisilicone proliferation			
n (%)^a^	(3.1)	(13.5)	0.048
Retinectomy			
180º, n (%)^a^	11 (17.2)	14 (37.8)	0.031
270º, n (%)^a^	4 (6.3)	3 (8.1)	0.705
360º, n (%)^a^	7 (10.9)	6 (16.2)	0.541
Tamponade^b^			
Air, n (%)^a^	1 (1.9)	3 (8.3)	0.119
SF6, n (%)^a^	0 (0)	2 (5.6)	
C3F8, n (%)^a^	14 (26.4)	6 (16.7)	
SO, n (%)^a,c^	38 (71.7)	25 (69.4)	
PPV (wide-field)			
25 g, n (%)^a^	50 (94.3)	21 (60.0)	< 0.001
23 g, n (%)^a^	3 (5.7)	14 (40.0)	
OR (95% CI)	11.1 (2.9–43.5)	Ref.	

*PPV* pars plana vitrectomy, *E-PPV* endoscopy-assisted PPV, *PVR* proliferative vitreoretinopathy, *Ref* reference category, *SO* silicone oil

^a^Proportions based on the total number of eyes included

^b^Tamponade information was missing for eleven eyes in the PPV only group and one eye in the endoscopy-assisted PPV group

^c^An additional 4 patients in the PPV-only group and 8 patients in the E-PPV group had used SO in a previous surgery

**Table 4:** Postoperative ocular characteristics and outcomes at last follow up

	Unadjusted analysis			Time-adjusted analysis^a^		
	PPV-only	E-PPV	*p* value	PPV-only	E-PPV	*p* value
BCVA (logMAR)						
Mean (95% CI)	1.42 (1.17–1.68)	1.34 (1.09–1.60)	0.201	1.44 (1.21–1.68)	1.32 (1.02–1.61)	0.518
IOP (mmHg)						
Mean (95% CI)	14.5 (12.6–16.5)	13.6 (11.2–16.1)	0.543	14.5 (12.5–16.5)	13.7 (11.3–16.1)	0.613
Phthisis						
n (%)	4 (7)	1 (2.7)		N/A	N/A	
OR (95% CI)	2.7 (0.3–25.3)	Ref.	0.362	3.0 (0.3–28.4)	Ref.	0.342
Follow-up (months)						
Mean (95% CI)	31.9 (25.2–38.6)	21.1 (16.4–25.8)	0.021	N/A	N/A	N/A
Number of surgeries						
Mean (95% CI)	2.7 (2.4–3.1)	4.1 (3.5–4.8)	< 0.001	2.6 (2.2–3.1)	4.3 (3.6–5.0)	< 0.001
Silicone oil removed						
n (%)^b^	18 (42.9)	18 (54.4)	0.315	N/A	N/A	0.099
OR (95% CI)	0.6 (0.2–1.6)	Ref.		0.4 (0.2–1.2)	Ref.	

*PPV* pars plana vitrectomy, *BCVA* best corrected visual acuity, *E-PPV* endoscopy-assisted PPV, *IOP* intraocular pressure, *IOL* intraocular lens, *N/A* not applicable, *Ref* reference category

^a^Adjusted for duration of follow-up

^b^Proportions based on eyes with silicon oil as tamponade (n_PPV-only_ = 42; n_E-PPV_ = 33)

**Fig. 1:**
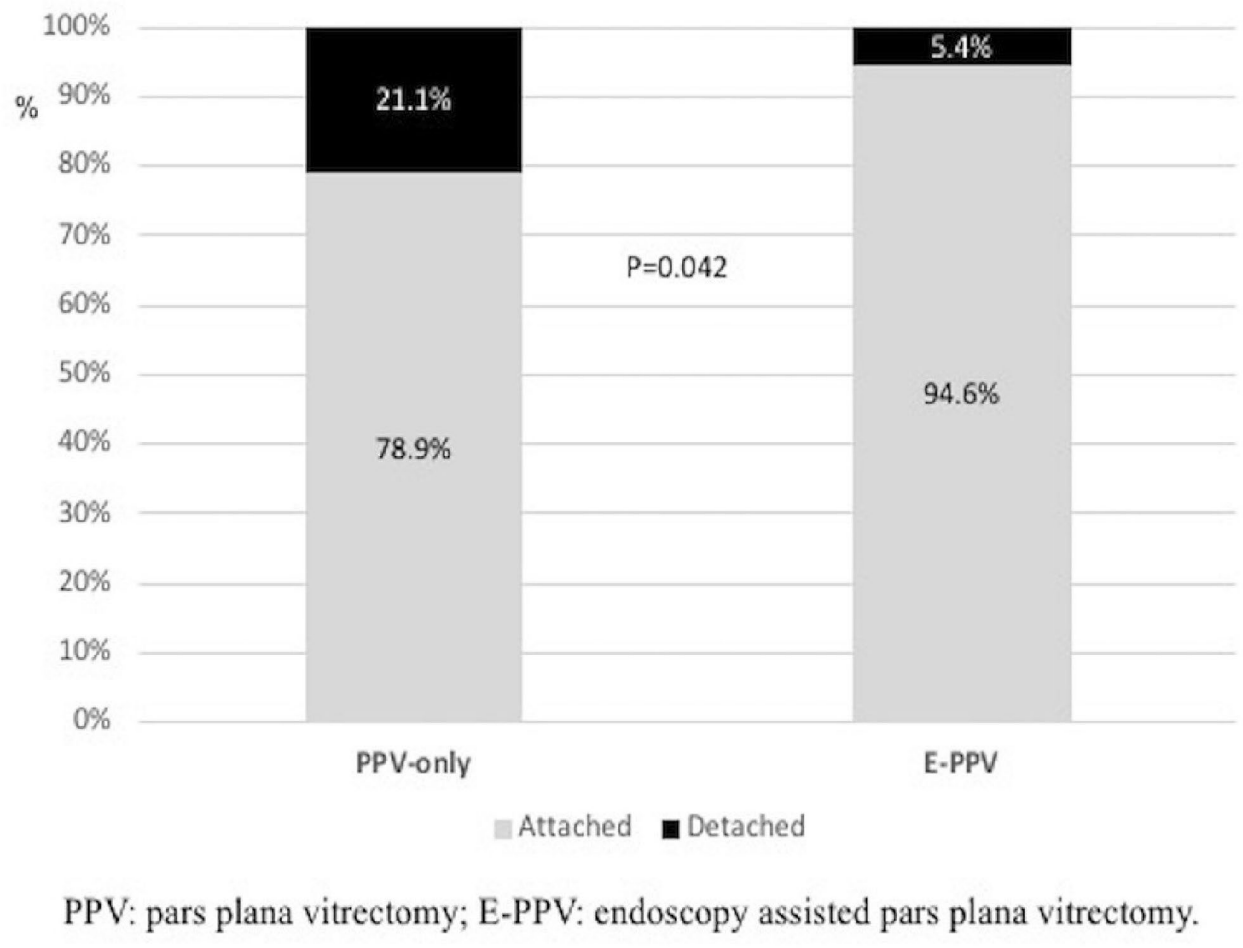
Retina final status. *PPV* pars plana vitrectomy, *E*-*PPV* endoscopy assisted pars plana vitrectomy

**Fig. 2:**
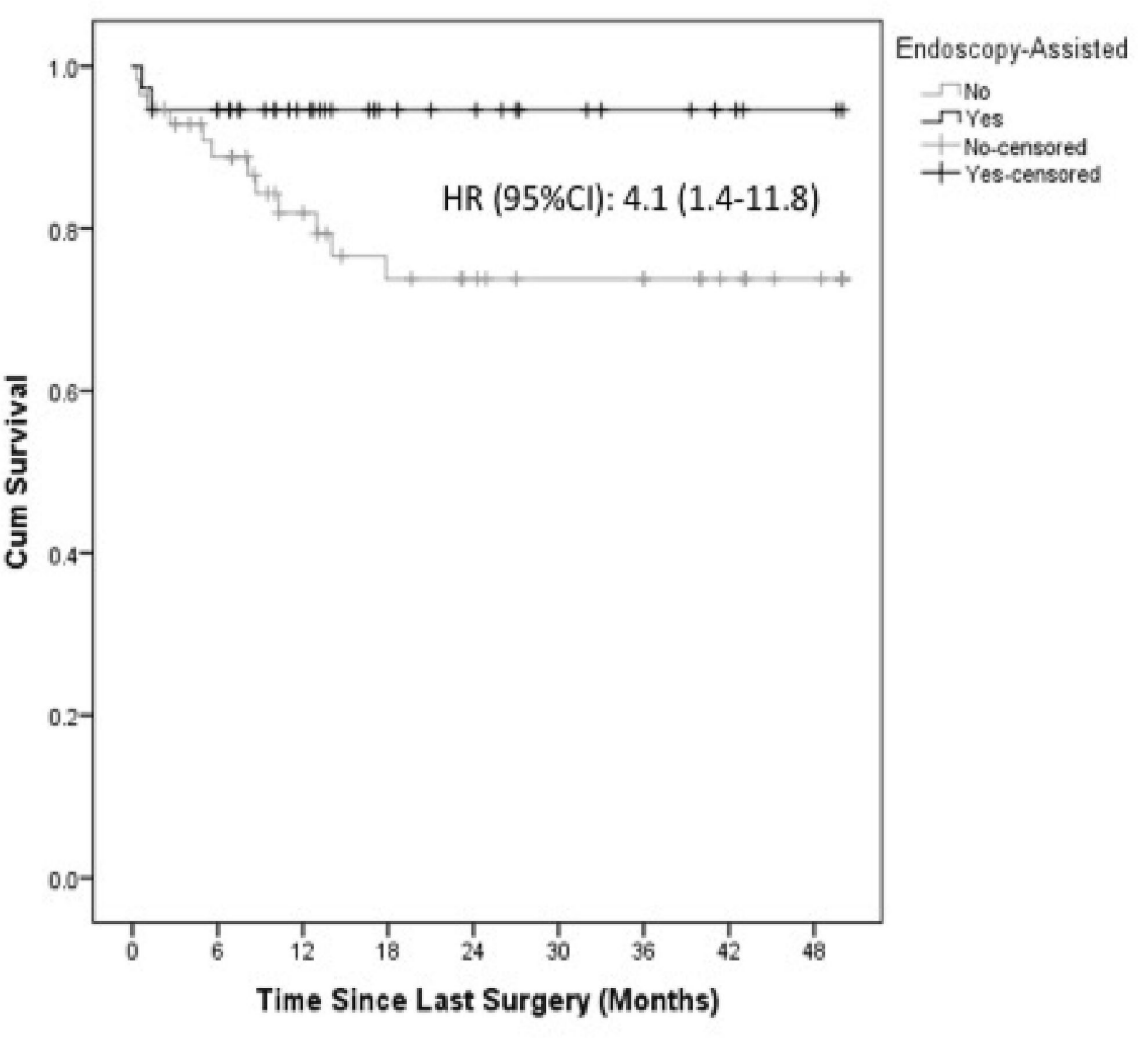
Time to retina redetachment

At the final follow-up, the reattachment rate was 78.9% in the PPV-only group and 94.6% in the E-PPV group (p = 0.037) (Fig. 1); in a sensitivity analysis excluding the group of patients with silicone oil-filled eyes, the reattachment rate at the final follow-up in the PPV-only and E-PPV groups was 87.9% and 100%, respectively. The Kaplan–Meier estimated time to redetachment was also significantly shorter in the PPV-only group, corresponding to a fourfold increase in the hazard rate (HR [95% CI] 4.1 [1.4–11.8]) (Fig. 2). Phthisis was 7% (n = 4) in the PPV-only group and 2.7% (n = 1) in the E-PPV group but did not reach statistical significance in either unadjusted analysis (OR [95% CI] 2.7 [0.3–25.3]) or after adjusting for follow-up duration (OR [95% CI] 3.0 [0.3–28.4]. No differences were found when comparing final VA or IOP between groups (Table 4).

## Discussion

Our results show that E-PPV may favor reattachment in RRD with advanced PVR. To the best of our knowledge, this is the first study comparing E-PPV vs. PPV alone.

The ability to have an undistorted view of the anterior VB as well as the retroiridial space and CB, enables the identification of different potential causes of redetachment that would not be detected with a wide-field visualization system during PPV alone and scleral indentation. Our intraoperative results revealed the following findings: undetected anterior traction, identification of anterior hyaloid contractions, contraction of the anterior edge of prior retinectomies, and identification of anterior PVR and CB traction. In addition, the ability to have better access to perform a more peripheral retinectomy, to peel multiple layers of the anterior hyaloid and the anterior ring of contraction at the Salzmann ligament area, and identify the need for capsular bag complex removal are also contributory factors that could explain the observed differences in reattachment rate (Additional file 1: Video S1). Nonetheless, the ability to identify the severity of anterior PVR at the CB region by examining the clinical appearance of the ciliary processes could have prognostic value.

We believe that the identification of these findings may have contributed to better anatomical results when E-PPV was performed. Boucher and Kuhn [8] reported the outcomes of 67 eyes after failed postprimary repair, and they identified the following findings with endoscopy: (1) persistent radial and/or circumferential vitreous adherences of the anterior vitreous base to the ciliary body, zonules, posterior lens capsule and iris; (2) persistent circumferential adherences inside the posterior part of the VB; (3) persistent anterior and posterior adherences; (4) reproliferation at the sclerotomy site; (5) CB detachment; (6) retinal break; (7) anterior neovascularization; and (9) subretinal proliferation. They concluded that the unique information provided by endoscopy correlated with experimental and pathological studies about the pathogenesis of anterior PVR. Yokohama et al. [12] evaluated the clinical outcomes for E-PPV in uncomplicated RRD, and the primary success rate was 98.4%. In our study, patients who underwent E-PPV showed a 94.6% reattachment rate, similar to uncomplicated RRD repair results, despite being a series of complex RRD with advanced PVR. Our cohort of patients differed from Boucher’s and Yokohama’s patients because they were recurrent RD with advanced PVR. Although our intraoperative findings were mostly related to anterior pathology, we also identified contraction of the edge of prior retinectomies in patients with PVR-B; furthermore, subretinal PVR was easily detected with endoscopy. These findings could explain why in cases of posterior PVR the reattachment rate was higher in the E-PPV group. In addition, as seen in one of our previous studies [15], during silicone oil removal the most common intraoperative endoscopy finding was contraction of previous retinectomy in patients with anterior and/or posterior PVR.

Effective drug management for PVR prevention or treatment remains an unmet need. The mainstay method for the management of PVR is surgery, but several studies have demonstrated that the interplay between cytokines, growth factors, matrix proteins and different cell types leads to the formation of pre, intra, and subretinal membranes. Different drugs and drug-delivery systems have been tested for the treatment of PVR. Pharmacologic agents such as anti-inflammatory drugs, antineoplasic/antiproliferative agents, antigrowth factors and antioxidants are currently being studied. The prevention of PVR will be a very important advancement in the prevention of redetachment; however, once the retina is redetached in PVR, it is imperative to treat this condition. E-PPV can help better identify and treat anterior PVR and potentially offers better anatomic results than PPV alone.

Limitations of this study are its retrospective nature, as well as the nonrandomized use of endoscopy. As such, due to the presence of potential confounders, the study findings should be interpreted as associations, and randomized prospective studies are necessary to draw causal inferences. There is a possible selection bias in the E-PPV group towards more complex RRDs, particularly in the early years of endoscopy adaptation, when it was applied when anterior PVR was clinically identified after failure in recurrent RD; in more recent years, endoscopy has been used routinely in combination with the 23-gauge platform in almost all pseudophakic recurrent redetachments, irrespective of the presence of clinically identifiable anterior PVR. This may explain the statistically higher presence of C5 and the higher number of surgical procedures used in the E-PPV group. The follow-up in the PPV-only group was longer than that in the E-PPV group because endoscopy only became available in 2013 at our institution. However, despite the different follow-up periods in each group, all patients were followed for a minimum of 6 months after the last surgery, which is an accepted standard follow-up time among vitreoretinal surgeons regarding postoperative retinal reattachment stability. In addition, a time-adjusted analysis was conducted in an effort to account for this difference. Finally, it is possible that some differences in postoperative outcomes were not identified as statistically significant due to a lack of statistical power; therefore, in interpreting statistically nonsignificant results, emphasis should be placed on clinical meaningfulness.

We included eyes with a minimum follow-up of 6 months after the last surgery regardless of the type of tamponade used in the last surgical procedure. The distribution of silicone oil-filled eyes was similar in both groups. Silicone oil was the long-term tamponade of choice in both groups in the following scenarios: monocular status, hypotony or patient choice. When excluding the group of patients with silicone oil-filled eyes, the attachment rate at final follow-up in the E-PPV vs. PPV-only groups was 100% vs. 87.9%. Although the study was not powered to detect this difference as statistically significant, the difference is clinically meaningful and merits further investigation in larger studies.

Unfortunately, few vitreoretinal surgeons have experience with E-PPV. This may be due to several factors, including a lack of image quality in two dimensions, difficulty in reaching the far periphery with current instrumentation and the small number of fellowship programs teaching this technique. However, recent technological improvements, such as the introduction of digitally enhanced visualization systems that increase the image resolution on a 4 K monitor and allow simultaneous viewing of both endoscopic and wide-field 3D images, should ease the challenges of learning E-PPV. This advancement, coupled with the positive results of the present study highlighting the potentially improved results using E-PPV in complex RRD, could reignite the interest of surgeons and industry investment in better technologies to address the anterior VB and retroiridial space.

## Conclusions

In this retrospective study, compared to PPV alone, endoscopy-assisted PPV was shown to be advantageous in achieving better reattachment rates in complex RRD with advanced PVR. Endoscopic visualization allowed a thorough examination and extensive anterior PVR and vitreous base dissection. Only one patient evolved to phthisis in the endoscopy group, despite a mean of 4 surgeries needed to achieve anatomical success.

## Supplementary information

**Additional file 1: Video S1.** Endoscopic intraoperative findings.

## Data Availability

The datasets used and/or analysed during the current study are available from the corresponding author on reasonable request.

## Abbreviations

BCVA: Best corrected visual acuity
CB: Ciliary body
CI: Confidence intervals
E-PPV: Endoscopy-assisted pars plana vitrectomy
ERM: Epiretinal membrane
ILM: Internal limiting membrane
IOL: Intraocular lens
IOP: Intraocular pressure
PPV: Pars plana vitrectomy
PVR: Proliferative vitreoretinopoathy
RD: Retinal detachment
RRD: Rhegmatogenous retinal detachment
VA: Visual acuity
VB: Vitreous base
